# Impact of pulmonary arterial systolic pressure on patients with mitral valve disease combined with atrial fibrillation

**DOI:** 10.3389/fcvm.2022.1047715

**Published:** 2023-01-09

**Authors:** Tie Zheng, Yichen Zhao, Qing Ye, Shuai Zheng, Fei Meng, Qiuming Hu, Haibo Zhang, Jie Han, Baiyu Tian, Junming Zhu, Jiangang Wang

**Affiliations:** Department of Cardiac Surgery, Beijing Anzhen Hospital, Capital Medical University, Beijing, China

**Keywords:** mitral valve, atrial fibrillation, pulmonary hypertension, Cox-Maze procedure, pulmonary artery systolic pressure

## Abstract

**Objective:**

To determine whether different changes of pulmonary artery systolic pressure (PASP) after surgeries have an impact on the long-term outcomes in patients with rheumatic and degenerative mitral valve (MV) disease and atrial fibrillation.

**Methods:**

Between 2004 and 2016, 1,188 patients with rheumatic and degenerative MV disease undergoing MV and Cox-Maze procedure were identified. Clinic outcomes, as well as rhythm state and echocardiography indices in long-term follow-up were recorded. Patients were grouped by the changes of PASP (persistently normal, persistently increased, increased, and decreased) from preoperative estimation to follow-up.

**Results:**

A complete echocardiography was performed at baseline and after 5 years. During follow-up, free of death and atrial fibrillation (AF) off antiarrhythmic drugs was 90 and 61%, 78 and 41% at 5 and 10 years, respectively. Survival rate was higher in patients with persistently normal and became worse in patients with persistently increased and increased PASP (log-rank 166.0, *P* < 0.0001). Moreover, the patients with persistently normal PASP had a lowest risk of recurrent AF (SHR: 0817; CI: 0.765–0.872; *P* < 0.0001) after considering death as a competing risk. A persistently normal PASP at follow-up and degenerative MV disease were associated with improved survival and sinus rhythm (SR) maintenance at multivariable Cox regression analysis (*P* < 0.05).

**Conclusion:**

Patients with degenerative MV disease or have persistently normal PASP during follow-up have better survival and SR maintenance rate than patients with either rheumatic MV disease or persistently abnormal PASP.

## Visual abstract

### Key questions

How are the impacts of pulmonary arterial systolic pressure on patients with mitral valve disease combined with atrial fibrillation?

### Key findings

A persistently normal PASP at follow-up and degenerative MV disease were associated with improved survival and sinus rhythm maintenance.

### Take-home message

For patients with rheumatic mitral diseases, more active interventions for valve disease and atrial fibrillation should be considered if PASP had obvious abnormity.

## Introduction

Pulmonary hypertension (PH) was known as one of serious complications of mitral valve (MV) disease, was originally reported in patients with rheumatic MV disease (RMVD) (1, 2). Recent studies have also showed that significant PH was present in patients with severe degenerative MV disease (DMVD) (3). More importantly, with more increasing evidences supporting the independent role of PH on negative long-term prognostic implications (4), the importance of PH deserves more attention.

Among patients with RMVD or DMVD, nearly 50% of them present with atrial fibrillation (AF), who needs to be treated with Cox-Maze procedure during cardiac operation according to the current guideline (5). Although Prof. Damiano found that the patients with RMVD or DMVD who underwent Cox-Maze procedure showed similar outcomes in terms of restoring sinus rhythm (SR) during a 41-month follow-up (6), some investigators suggested that the Cox-Maze procedure was less effective in patients with RMVD (7). Therefore, our study was aimed at to characterizing the clinical features and the significance of PH regarding prognosis in a large cohort of patients with RMVD or DMVD after Cox-maze procedure.

## Materials and methods

### Patient selection

This study was approved by the institutional review board (20180723) of Beijing Anzhen Hospital, Capital Medical University. Patients with MV disease and concomitant AF requiring MV and Cox maze procedure were identified with a review of our center’s medical records from 2004 to 2016. Among 1,689 patients initially screened for inclusion, 1,188 patients with rheumatic and degenerative MV disease who discharged alive were included in our study cohort. All patients with non-rheumatic/non-degenerative mitral valve (MV) disease were excluded, which encompassed patients with MV disease secondary to annular dilation or calcification, infectious endocarditis, ischemic heart disease, congenital heart disease and prosthetic valve dysfunction. Patients who underwent tricuspid valve surgeries before were also excluded in this study.

Rheumatic MV disease was determined by the history of acute rheumatic fever. Echocardiographic and intraoperative findings of leaflet thickening, nodularity, and commissural fusion, resulting in narrowing of the valve, were considered as rheumatic MV disease. According to the guidelines, patients could be diagnosed as long-standing persistent AF when their rhythm kept AF for more than 12 months. All patients referred in this study gave informed consent.

### Surgical procedures

MV repair and replacement were performed using standard cardiopulmonary bypass. All the patients underwent a Cox-Maze procedure as described in our previous study, with the bipolar radiofrequency clamp (8). Tricuspid valve (TV) annuloplasty was subsequently performed for patients with moderate or higher TR or tricuspid annular diameter (TAD) more than 40 mm. Details on Cox-maze procedure were shown in Supplementary material.

### Echocardiography

**FIGURE 1:**
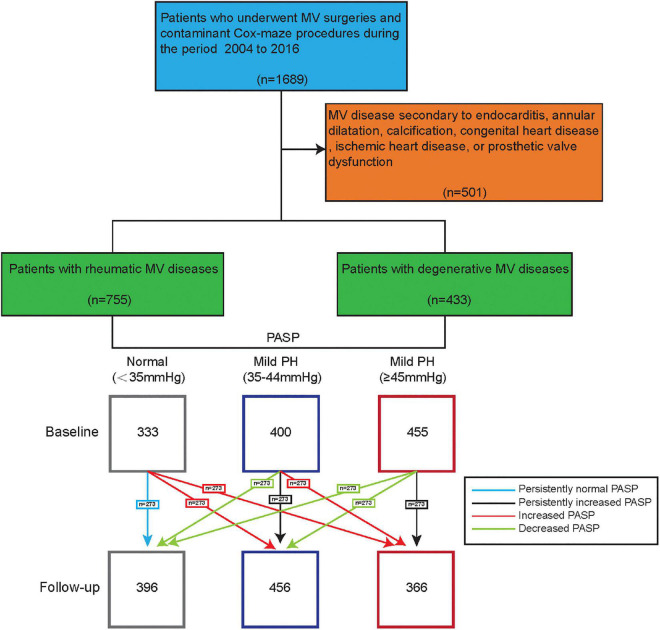
Individual changes of pulmonary artery systolic pressure (PASP) from baseline to 5-year follow-up.

All echocardiography examinations were conducted in accordance with current guidelines (9). Based on the preoperative PASP, patients were divided into three groups: normal PH, mild PH (35–44 mm Hg), and moderate-to-severe PH (≥ 45 mm Hg) (10). At follow-up, based on changes of PASP changes from baseline to follow-up evaluated by echocardiography, patients were than divided into four groups: (1) persistently normal PASP; (1) decreased PASP (PASP decreased at least one category from baseline); (3) increased PASP (PASP increased least one category from baseline); and (4) persistently increased PASP (Figure 1). Echocardiographic images including standard 2D and Doppler were completed in the parasternal and apical views by trained cardiac sonographers. The measurement of PASP was based on the modified Bernoulli equation which composed of the trans-tricuspid Doppler signal and RA pressure. PASP were acquired by averaging values of at least three times measurements. RA pressure was evaluated based on inferior vena cava diameter (IVCd) and the collapsible index of inferior vena cava (IVC). IVCd was measured from the longitudinal subxiphoid view with M-mode, at end-expiration and end-inspiration, respectively. Then the collapsible index of IVC was calculated [collapsible index of IVC = (IVCd at end-expiration - IVCd at end-inspiration)/IVCd at end-expiration]. After acquiring the IVCd and collapsible index, RA pressure could be divided into three grades (3, 8, and 15 mmHg) according to the ASE recommendations (11).

### Data collection and follow-up

The patients’ clinical profiles and postoperative outcomes were extracted from the hospital medical records. Patients were followed at 3 months, 12 months, and then every year. Recurrent arrhythmia was evaluated by a 24-h Holter. According to the HRS guidelines, late recurrence could be diagnosed when patients meet the following conditions: atrial arrhythmia including AF, atrial flutter or atrial tachycardia which could last more than 30 s and occurred after 3 months following surgery. Sinus rhythm maintenance was defined when the cardiac rhythm was sinus rhythm at each echocardiography evaluation during follow-up. Long-term outcomes were acquired by questionnaires, physician clinical visits during follow-up.

### Medicine treatment during follow-up

Class I or III antiarrhythmic drugs (AADs) and warfarin were used routinely postoperatively unless contraindicated. If SR could be maintained for 3 months postoperatively, patients would discontinue AADs. Once patients were diagnosed as recurrent AF, they would be informed to restart AADs. Considering the importance of rate control in AF, patients with persistent AF also took other agents, including beta-blockers, digoxin, non-dihydropyridine calcium-channel blockers (CCBs) and so on. Patients’ hemodynamic features, symptoms and heart failure would be taken into consideration when cardiologist determine which agents to use.

The international normalized ratio (INR) were requested to maintain between 2.0 and 2.5 when patients taken oral anticoagulation in the first 6 months after surgery, and those who had mechanical prosthesis or persistent AF were requested to take oral anticoagulation for life.

### Statistical analysis

Categorical variables were presented as frequencies and percentages. Normal distributed continuous variables were presented as mean ± standard deviation (SD). Data among 3 or 4 groups were compared with one way analysis of variance, or Kruskal- Wallis test. Survival analysis were completed with Kaplan- Meier analysis and Cox regression models. The incidence of recurrent AF were compared with competing risk model. A PASP cut-off value for even-free survival were acquired with the analysis of time-dependent receiver operating characteristic curve. Statistical analyses were performed using R 3.6.2 or Stata/SE version 15.

## Results

### Baseline characteristics

Table 1 showed the clinical and echocardiographic features of the study population (1,188 patients), as shown in Table 1. The mean age of all the patients was 48 (39–57) years; Patients with rheumatic MV disease accounted for 64% of cases, and 36% of patients presented with DMVD. Significant mitral stenosis (mitral valve area: 1.02 ± 0.31 cm^2^) were observed among all patient with RMVD, and 42% of which showed over moderate mitral regurgitation preoperatively. Patients with RMVD who underwent mitral valve replacement accounted for 14%, and 86% of whom received mitral valve repair. Similarly, 11% of patients with DMVD underwent mitral valve replacement, and 89% of them received mitral valve repair. No significant differences in terms of procedures were observed between the two groups.

**TABLE 1:** Baseline characteristics of patients.

		PASP at baseline			
	All patients *n* = 1,188	Normal (<35 mmHg), *n* = 333	Mild PH (35–44 mmHg), *n* = 400	Moderate-to-severe PH (≥45 mmHg), *n* = 455	*P*-value
Age, years (IQR)	48 (39–57)	49 (39–58)	48 (39–57)	48 (37–57)	0.799
Male (%)	719 (60.52)	185 (55.6)	251 (62.7)	283 (62.2)	0.091
BSA, m^2^ (IQR)	1.7 (1.6–1.8)	1.7 (1.6–1.8)	1.7 (1.6–1.8)	1.7 (1.6–1.8)	0.135
NYHA class (IQR)	3 (2–3)	3 (2–3)	3 (2–3)	3 (2–3)	0.569
CHA2DS2-Vasc score ≥ 2 (%)	130 (101)	35 (11)	46 (12)	49 (11)	0.902
EuroSCORE (IQR)	0 (0–1)	0 (0–1)	0 (0–1.75)	0 (0–2)	0.774
RMVD (%)	755 (64)	204 (61)	239 (60)	312 (69)*,^††^	0.017
DMVD (%)	433 (36)	129 (39)	161 (40)	143 (31)*,^††^	0.017
AF duration, months (IQR)	5 (3–10)	3 (2–3)	3 (2–3)	3 (2–3)	0.470
Paroxysmal AF	102 (9)	39 (11)	33 (9)	30 (7)	0.575
PASP, mmHg (IQR)	41 (34–49)	31 (28–33)	40 (38–43)***	51 (48–54)***,†††	<0.001
PASP at follow-up, mmHg (IQR)	37 (32–47)	36 (31–46)	37 (32–47)*	40.0 (32–50)***,^†^	<0.001
LAD, mm (IQR)	57 (47–66)	56.0 (47–66)	55.0 (45–65)	58 (48–67)^††^	0.029
LVEF, % (IQR)	60 (54–65)	59 (53–65)	59 (54–65)	60 (55–66)	0.244
RA area, mm^2^ (IQR)	20 (17–23)	20 (17–23)	20 (17–23)	20 (17–23)	0.355
TAPSE, mm (IQR)	14 (12–16)	14 (12–16)	14 (12–16)	14 (13–16)	0.353
TAD, cm (IQR)	37 (34–40)	37 (34–40)	37 (34–40)	37 (34–40)	0.322
TR grade (IQR)	1 (1–2)	1 (1–2)	1 (1–2)	1 (1–2)	0.201
MV repair (%)	546 (46)	188 (57)	178 (45)**	180 (40)***	<0.001
MV replacement (%)	642 (54)	145 (44)	222 (56)**	275 (60)***	<0.001
CABG (%)	58 (5)	18 (6)	24 (6)	16 (4)	0.212
TV repair (%)	563 (47)	143 (43)	208 (52)*	253 (56)***	0.002
CPB, min (IQR)	101 (75–127)	99 (75–125)	99 (73–126)	103 (77–131)	0.230
ACT, min (IQR)	63 (45–85)	61 (45–83)	62 (45–84)	64 (46–87)	0.221

ACT, aortic clamp time; AF, atrial fibrillation; BSA, body surface area; CABG, coronary artery bypass graft; CPB, cardiopulmonary bypass; DMVD, degenerative mitral valve disease; IQR, interquartile range; LAD, left atrial diameter; LVEF, left ventricle ejection fraction; MV, mitral valve; NYHA, New York Heart Association; PASP, pulmonary artery systolic pressure; PH, pulmonary hypertension; RMVD, rheumatic mitral valve disease; TAD, tricuspid annulus diameter; TR, tricuspid regurgitation; RA, right atrial; TAPSE, tricuspid annular plane systolic excursion. Echocardiographic information was postoperative before discharge.

*P < 0.05, **P < 0.01, and ***P < 0.001 vs. normal PH; ^†^*P* < 0.05, ^††^*P* < 0.01, and ^†††^*P* < 0.001 vs. mild PH.

### Five-year echocardiographic follow-up: Pulmonary artery systolic pressure

Considering the whole population, PASP significantly decreased at 5-year follow-up (from 42 ± 9 to 39 ± 9 mmHg, *P* < 0.001); however, it remained almost unchanged in patients with RMVD (from 41 ± 9 to 42 ± 9 mmHg, *P* = 0.512), while it significantly decreased in patients with DMVD (from 42 ± 9 to 35 ± 6 mmHg, *P* < 0.001). The baseline clinical and echocardiographic features of patients who were grouped based on the PASP changes during follow-up were shown in Table 2. Compared with patients showed normal PASP persistently, other three groups patients had higher prevalence of etiology of RMVD, more MV replacement and TV repair procedure, and higher baseline PASP values.

**TABLE 2:** Baseline characteristics of patients according to changes of PASP at follow-up.

	PASP at follow-up				
	Persistently normal, *n* = 207	Decreased, *n* = 189	Increased, *n* = 456	Persistently increased, *n* = 336	*P*-value
Age, years (IQR)	49 (14–58)	47 (37–56)	48 (38–47)	51 (39–58)	0.086
Male (%)	119 (58)	109 (58)	274 (60)	217 (65)	0.159
BSA, m2 (IQR)	1.7 (1.6–1.8)	1.7 (1.6–1.9)	1.7 (1.6–1.8)	1.7 (1.6–1.8)	0.888
NYHA class (IQR)	3 (2–3)	3 (2–3)	3 (2–3)	3 (2–3)	0.972
CHA2DS2-Vasc score ≥ 2 (%)	25 (12)	17 (9)	47 (10)	41 (12)	0.627
EuroSCORE (IQR)	0 (0–1)	0 (0–1)	0 (0–2)	0 (0–1)	0.294
RMVD (%)	80 (39)	103 (55)*	325 (71**,^††^	247 (74**,^††^	*P* < 0.001
DMVD (%)	127 (61)	86 (46)*	131 (29)**,^††^	89 (27)**,^††^	*P* < 0.001
AF duration, months (IQR)	3 (2–3)	3 (2–3)	3 (2–3)	3 (2–3)	0.766
PASP, mmHg (IQR)	32 (29–35)	37 (32–47)**	45 (39–51)**, ^††^	46 (39–51)**, ^††^	*P*< 0.001
PASP at follow-up, mmHg (IQR)	32 (29–35)	37 (34–47)**	35 (30–43)**,^††^	48 (40–52)**,^††,‡‡^	*P*< 0.001
LAD, mm (IQR)	57 (48–67)	55 (48–66)	56 (46–65)	57 (47–66)	0.803
LVEF, % (IQR)	59 (52–65)	61 (55–65)	59 (54–65)	60 (54–65)	0.492
RA area, mm2 (IQR)	20 (17–23)	20 (17–23)	20 (17–23)	21 (17–23)	0.855
TAPSE, mm (IQR)	14 (12–23)	15 (13–16)	14 (13–16)	14 (12–16)	0.286
TAD, cm (IQR)	37 (34–40)	38 (34–41)	37 (34–40)	37 (34–40)	0.554
TR grade (IQR)	1 (1–2)	1 (1–2)	1 (1–2)	1 (1–2)	0.559
MV repair (%)	168 (81)	95 (50)**	180 (40)**, ^†^	103 (31)**, ^††, ‡^	*P*< 0.001
MV replacement (%)	39 (19)	94 (50)**	276 (61)**, ^†^	223 (69)**, ^††, ‡^	*P*< 0.001
CABG (%)	8 (5)	19 (4)	13 (5)	18 (6)	0.776
TV repair (%)	72 (35)	93 (49)*	237 (52)**	202 (60)**,^†,‡^	*P*< 0.001
CPB, min (IQR)	100 (74–125)	98 (74–123)	105 (76–127)	97 (74–131)	0.591
ACT, min (IQR)	62 (45–83)	61 (45–81)	65 (42–85)	60 (45–87)	0.567

ACT, aortic clamp time; AF, atrial fibrillation; BSA, body surface area; CABG, coronary artery bypass graft; CPB, cardiopulmonary bypass; DMVD, degenerative mitral valve disease; IQR, interquartile range; LAD, left atrial diameter; LVEF, left ventricle ejection fraction; MV, mitral valve; NYHA, New York Heart Association; PASP, pulmonary artery systolic pressure; PH, pulmonary hypertension; RMVD, rheumatic mitral valve disease; TAD, tricuspid annulus diameter; TR, tricuspid regurgitation; RA, right atrial; TAPSE, tricuspid annular plane systolic excursion. Echocardiographic information was postoperative before discharge.

*P < 0.05, and **P < 0.001 vs. persistently normal PH; ^†^*P* < 0.05, and ^††^*P* < 0.001 vs. decreased PH; ^‡^*P* < 0.05, and ^‡‡^*P* < 0.001 vs. increased PH.

### Survival and rhythm status during follow-up

**FIGURE 2:**
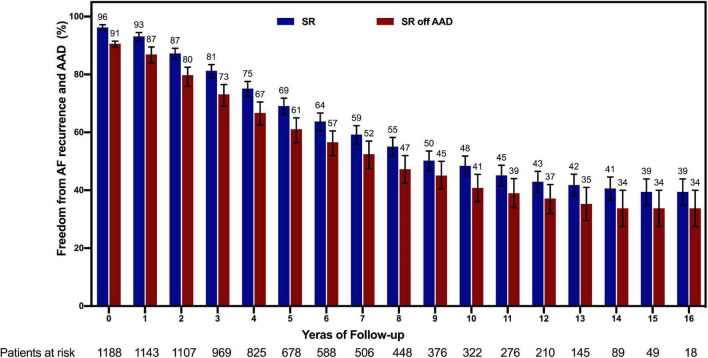
Follow-up showing freedom from recurrence of AF both on and off AADs with 95% confidence intervals. AF, atrial fibrillation; AAD, antiarrhythmic drug; SR, sinus rhythm.

During a mean follow-up of 8 years (4–12 years), a total of 173 deaths were observed, of which 106 were of cardiovascular origin. Overall survival of the total population was 90% (95% CI, 92–98) at 5 years and 78% (95% CI, 75–81) at 10 years. During follow-up, 76 patients had reoperation, 35 had a stroke, and 7 patients had implanted pacemaker. In total population, AF freedom on and off AAD was 69% (95% CI, 66–72) and 61% (95% CI, 57–65) at 5 years; 48% (95% CI, 45–52) and 41% (95% CI, 36–46) at 10 years, respectively (Figure 2).

Etiology, MV procedure, and PASP changes at follow-up were significant univariable predictors for both death and AF recurrence. Furthermore, LAD, PASP at baseline, and TV repair were significant univariable predictors of AF recurrence. While etiology and PASP changes at follow-up in death-free and AF recurrence free survival remained significant after multivariable adjustment (Table 3). Details of univariate regression were shown in Supplementary material.

**TABLE 3:** Multivariable time-dependent Cox regression model.

	HR (95% CI)	*P*-value
**Multivariable model for death**		
DMVD vs. RMVD	0.50 (0.29–0.87)	0.015
LAD (per 1 SD)	0.95 (0.82–1.10)	0.523
LVEF (per 1 SD)	0.96 (0.83–1.11)	0.542
MV procedure (Replacement vs. repair)	1.45 (0.91–2.32)	0.120
TAPSE (per 2 mm)	1.06 (0.91–1.22)	0.474
RA area (per 5 cm^2^)	1.00 (0.84–1.18)	0.980
TAD (per 4 mm)	1.07 (0.92–1.26)	0.379
TV repair (yes vs. no)	0.90 (0.66–1.23)	0.499
Increased or persistently increased PASP vs. persistently normal PASP	5.81 (2.53–13.36)	<0.001
Increased or persistently increased PASP vs. decreased PASP	5.36 (2.37–12.13)	<0.001
**Multivariable model for AF**		
DMVD vs. RMVD	0.69 (0.50–0.96)	0.027
PASP (per 15 mmHg)	0.96 (0.81–1.14)	0.640
EUROScore (high risk vs. moderate or mild risk)	0.75 (0.55–1.02)	0.070
LAD (per 1 SD)	0.99 (0.90–1.08)	0.744
LVEF (per 1 SD)	0.98 (0.90–1.07)	0.686
MV procedure (Replacement vs. repair)	0.85 (0.62–1.15)	0.291
TAPSE (per 2 mm)	1.08 (0.99–1.18)	0.095
RA area (per 5 cm^2^)	1.03 (0.92–1.14)	0.633
TAD (per 4 mm)	1.06 (0.96–1.17)	0.065
TV repair (yes vs. no)	0.09 (0.90–1.32)	0.392
Increased or persistently increased PASP vs. persistently normal PASP	51.32 (12.68–207.71)	<0.001
Increased or persistently increased PASP vs. decreased PASP	1.28 (0.78–0.91)	0.081

AF, atrial fibrillation; CI, confidence interval; DMVD, degenerative mitral valve disease; HR, hazard ratio; LAD, left atrial diameter; LVEF, left ventricular ejection fraction; MV, mitral valve; PASP, pulmonary artery systolic pressure RA, right atrium; RMVD, rheumatic mitral valve disease; TAD, tricuspid annular diameter; TV, tricuspid valve; SD, standard deviation.

**FIGURE 3:**
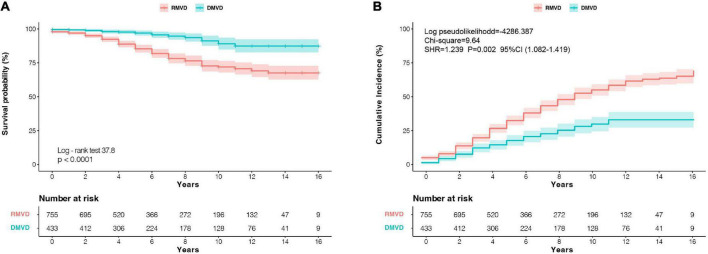
**(A)** Kaplan–Meier survival analysis, and **(B)** cumulative incidence analysis of probability of recurrence of AF according to the MV etiology. CI, confidence interval; DMVD, degenerative mitral valve disease; RMVD, rheumatic mitral valve disease; SHR, sub-hazard ratio.

**FIGURE 4:**
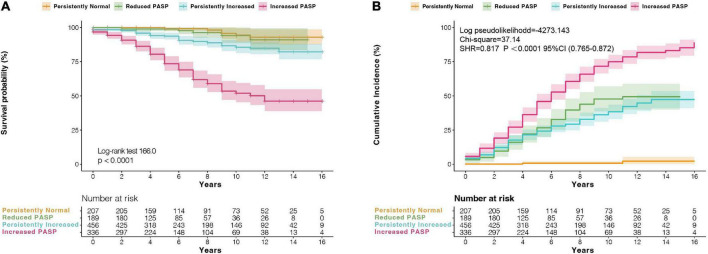
**(A)** Kaplan–Meier survival analysis, and **(B)** cumulative incidence analysis of probability of recurrence of AF according to changes of pulmonary artery systolic pressure (PASP) at follow-up. CI, confidence interval; SHR, sub-hazard ratio.

Patients with DMVD had the significantly better death-free survival compared with patients with RMVD (log-rank 37.8, *P* < 0.0001, Figure 3A). Meanwhile, compared with RMVD, the patients with DMVD also had a lower risk of AF recurrence (SHR: 1.239; CI: 1.082–1.419; *P* = 0.002, Figure 3B) after considering death as a competing risk. Moreover, when patients were stratified according to the PASP changes at 5-year follow-up, a higher survival rate was observed among patients with persistently normal and decreased PASP (log-rank 166.0, *P* < 0.0001, Figure 4A). Likewise, the patients with persistently normal PASP had a lowest risk of recurrent AF (SHR: 0.817; CI: 0.765–0.872; *P* < 0.0001, Figure 4B) in the competing model that death was seen as a competing event.

**FIGURE 5:**
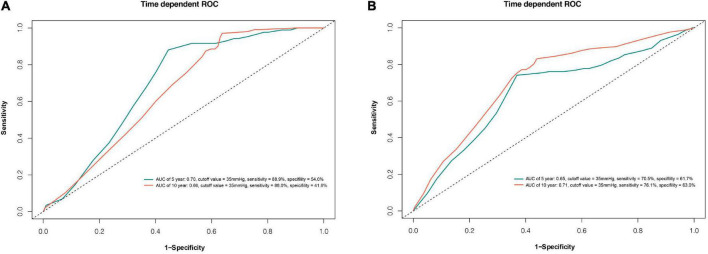
Time-dependent receiver operating characteristic (ROC) curve for pulmonary artery systolic pressure (PASP) at follow-up with optimal cut-off point best predicting death **(A)** and AF recurrence **(B)**. AUC, area under curve.

The Time-dependent ROC curve indicated that PASP of 35 mmHg at follow-up discriminated predicting 5-(sensitivity 89%, specificity of 54%, AUC 0.70), and 10-(sensitivity 88%, specificity of 42%, AUC 0.66) year death-free survival patients, respectively (Figure 5A). Similarly, a value of 35 mmHg at follow-up discriminated predicting 5-(sensitivity 71%, specificity of 62%, AUC 0.65), and 10-(sensitivity 76%, specificity of 63%, AUC 0.71) year recurrent AF-free survival patients, respectively (Figure 5B).

**FIGURE 6:**
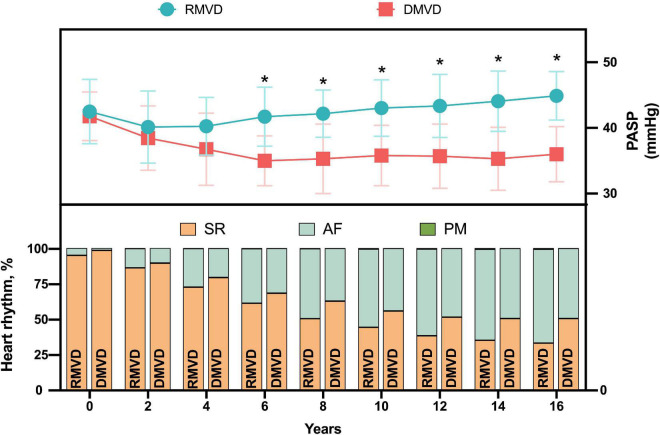
Heart rhythm according to the changes of PASP during follow-up in patients with MV disease. AF, atrial fibrillation; DMVD, degenerative mitral valve disease; PASP, pulmonary artery systolic pressure; PM, pacemaker; RMVD, rheumatic mitral valve disease; SR, sinus rhythm. *P < 0.05 vs. DMVD.

Figure 6 demonstrated PASP was significantly higher in patients with RMVD, which was accompanied by a lower SR maintenance rate.

## Comment

To our knowledge, our study is the first one that demonstrates the association between MV etiology and PASP with improved survival in patients with MV disease with AF. Our results illustrated that the improvement of PH during follow-up may directly affect the clinical outcomes of patients after MV procedure and Cox-Maze procedure. These observations also highlight the important contributions of MV etiology and PH in maintaining clinical compensation.

AF is a common sequela of MV disease that is frequently present in patients referred to MV surgery (12, 13). MV disease may lead to the development of AF *via* affecting LA volume and pressure overload, causing progressive atrial fibrosis, LA structural remodeling and electrical remodeling (14). The Cox-Maze procedure, using a bipolar radiofrequency device, has been shown to be the most effective surgical treatment of AF (15, 16). However, the outcomes of the Cox-Maze procedure in treating AF associated with different etiology of MV disease remained controversial. Labin et al. reported that compared with patients with DMVD with a longer duration of AF and larger LAD, despite representing a sicker patient population, patients with RMVD may equally benefit from the Cox-Maze procedure up to 5 years of follow-up (6). Nevertheless, our results showed significantly worse event-free survival in patents with RMVD during a 15-years follow-up.

Hemodynamic evaluation of patients with DMVD associated PH reveals that the increase of pulmonary arterial pressure of these patients were positioned in the incipient stage, which were mainly caused by the increase of left atrial pressure (17). In this stage, the resistance of pulmonary vessel showed normal function. Patients may show a high transpulmonary pressure gradient but this status could usually be reversed after successful mitral interventions (18, 19). Although, the pathology mechanism of pulmonary vessel among patients with DMVD could be affected by a lot of factors that are not fully understood yet (20), the fact is that many patients with severe DMVD in the real world have not been observed any elevations of pulmonary artery pressure (21).

However, in patients with RMVD (long-standing course MV disease), PASP had a lower possibility of decreasing PASP though their LAP and PH may maintain normal postoperatively (2, 22), which indicated that the irreversibility of PH caused by pulmonary vascular remodeling may led to worse clinical outcomes. The above persistence of significant PH after surgery in patients with RMVD may be the result of persistent microvascular changes.

As with other retrospective studies, several limitations of this study are as follows. First, PH was defined as mean PAP of more than 20 mm Hg measured with right heart catheterization (RHC). RHC is an invasive procedure with associated risk, which limits the utility for routine evaluation of PH during follow-up. However, echocardiography has been an effective and relatively precise tool to evaluate PASP since the correlation was found between RHC and echocardiography, and 35 mmHg is the most used threshold value to estimate PH *via* echocardiography (23). Second, the occurrence of missing data is also one of the limitations. Not all patients had rhythm status and echocardiography available for each time point.

## Conclusion

Our data indicated that patients with DMVD or have persistently normal PASP during follow-up have better survival and SR maintenance, while patients with RMVD or have an abnormal PASP during follow-up have worse outcome. For patients with rheumatic mitral diseases, more active surgical interventions should be considered if PASP had obvious abnormity.

## Data availability statement

The raw data supporting the conclusions of this article will be made available by the authors, without undue reservation.

## Ethics statement

This study has been approved by the Institutional Review Board (20180723) at Beijing Anzhen Hospital, Capital Medical University. The patients/participants provided their written informed consent to participate in this study.

## Author contributions

All authors listed have made a substantial, direct, and intellectual contribution to the work, and approved it for publication.
