# Supplementary figures and images for: Impact of pulmonary arterial systolic pressure on patients with mitral valve disease combined with atrial fibrillation
Source: Front Cardiovasc Med. 2023 Jan 9;9:1047715. doi: 10.3389/fcvm.2022.1047715 (PMC9868267; doi:10.3389/fcvm.2022.1047715)

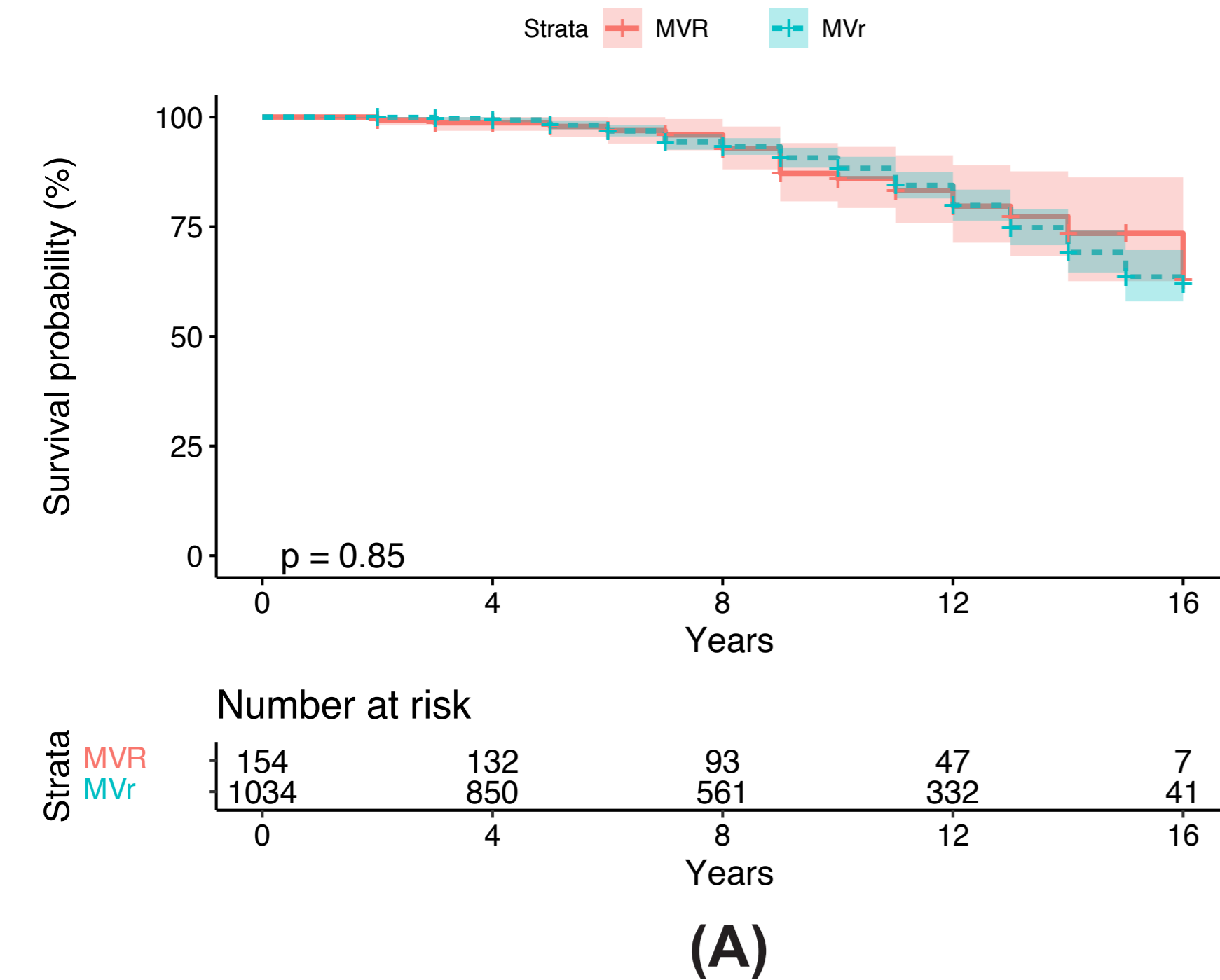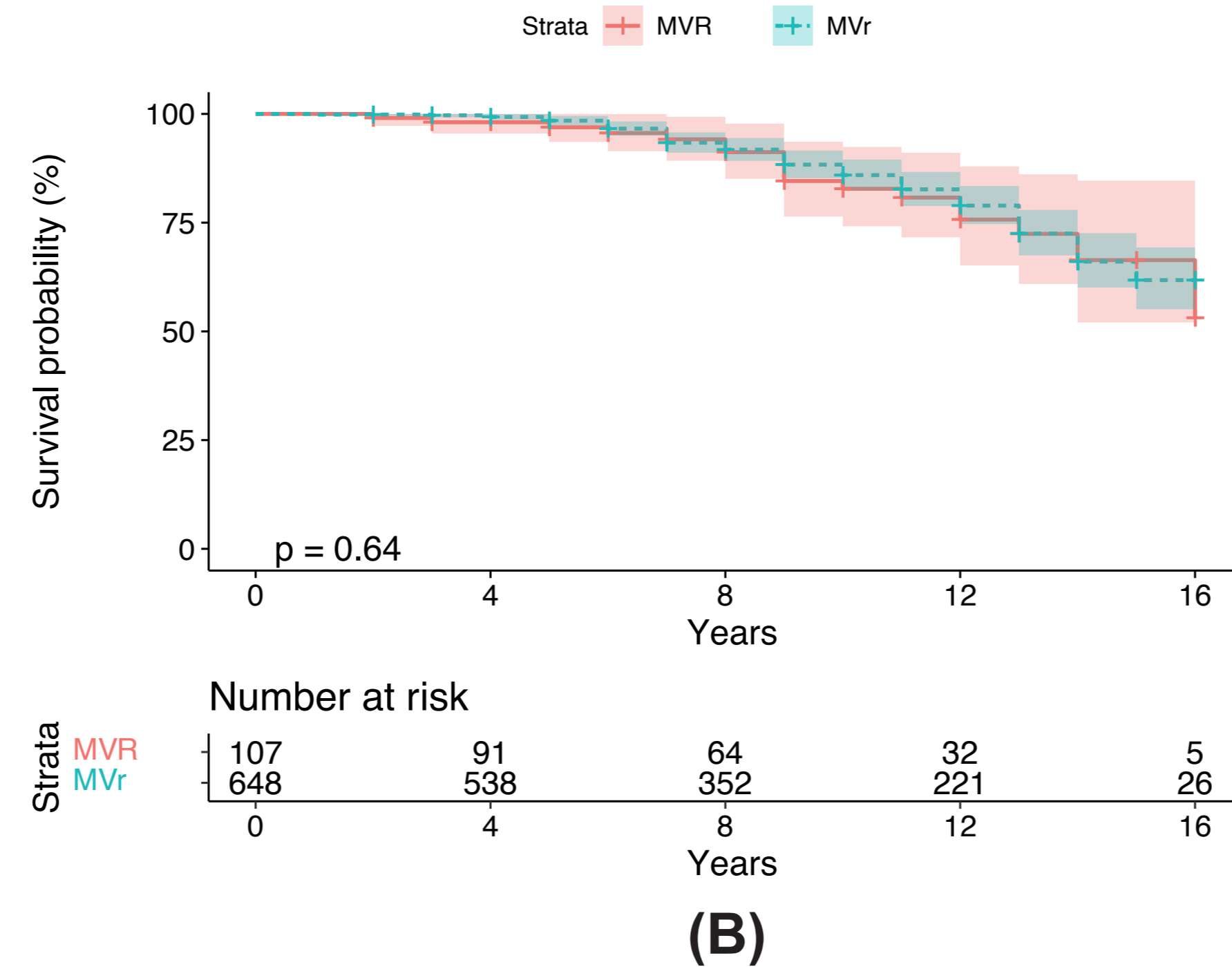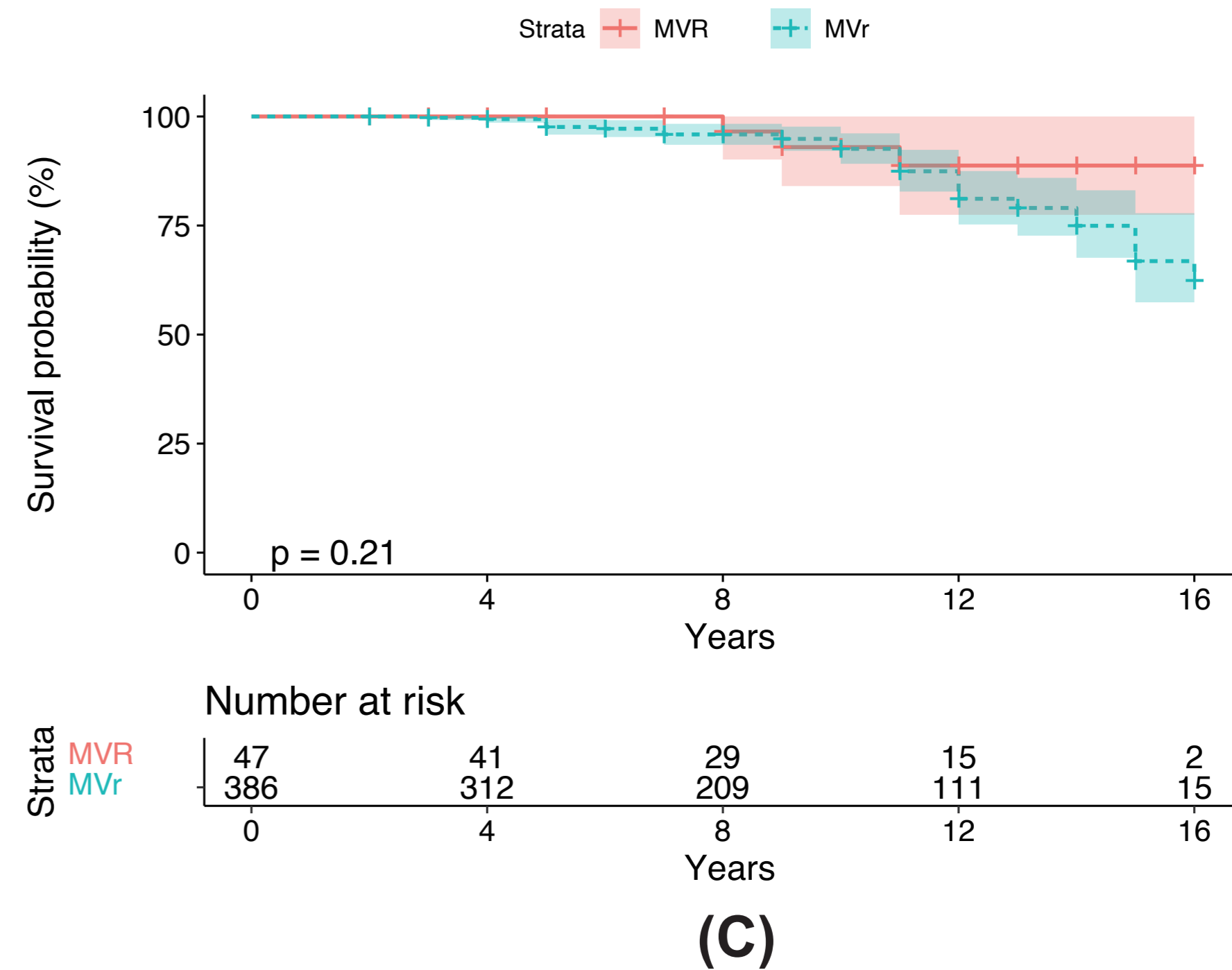

Supplement: Supplementary file 1 [file Image_1.pdf]
