# Supplementary material for: Impact of pulmonary arterial systolic pressure on patients with mitral valve disease combined with atrial fibrillation
Source: Front Cardiovasc Med. 2023 Jan 9;9:1047715. doi: 10.3389/fcvm.2022.1047715 (PMC9868267; doi:10.3389/fcvm.2022.1047715)
Supplement: Supplementary file 2 [file Data_Sheet_1.docx]

**Supplemental Method**

***Cox-maze procedure***

All procedures were performed using routine cardiopulmonary bypass with bicaval and aortic cannulation under moderate hypothermia. The procedure was carried out with the bipolar Cardioablate (Medtronic) or Atricure clamp (Atricure). After cardioplegic arrest, a left atrial incision was performed through the interatrial groove. The left atrial appendage was either amputated and sutured afterward, or a circumferential radiofrequency lesion was created around its base and the oriﬁce oversewn from inside the atrium. In addition to the incision in the interatrial groove, isolation of the right pulmonary veins was completed by a circular ablation line. The left pulmonary veins were encircled and a connecting line was performed between both islands of pulmonary veins on the roof, as near to the left atrial roof as possible to avoid injury to the esophagus. An ablation line from the left pulmonary veins to the posterior mitral annulus was then performed with caution so as not to injure the circumﬂex coronary artery. Cavotricuspid isthmus ablation was then performed to achieve a bidirectional conduction block. Division of the ligament of Marshall was performed in all patients. In the right atrial lesions, the following lesions, other than cavotricuspid isthmus ablation, were added in the right atrium: excision of right atrial appendage; superior vena cava to inferior vena cava; lateral free-wall lesion complete to anterior-medial tricuspid valve annulus; and medial free-wall lesion complete to anterior-medial tricuspid valve annulus.

**Supplemental Table（ Univariable Cox regression model）**

| **Variable** | **Death** | | **Atrial fibrillation** | |
| --- | --- | --- | --- | --- |
|  | **HR (95% CI)** | **P-value** | **HR (95% CI)** | **P-value** |
| DMVD vs. RMVD | 0.30 (0.20-0.45) | ＜0.0001 | 0.45 (0.36-0.56) | ＜0.0001 |
| Age (per 1 SD) | 1.06 (0.91-1.24) | 0.434 | 1.03 (0.94-1.13) | 0.570 |
| Sex (Male) | 1.10 (0.82-1.50) | 0.519 | 1.01 (0.84-1.22) | 0.900 |
| BSA (per 1 SD) | 0.98 (0.85-1.14) | 0.823 | 1.02 (0.93-1.12) | 0.681 |
| NYHA class ＞II | 1.18 (0.85-1.14) | 0.332 | 1.07 (0.88-1.31) | 0.482 |
| Paroxysmal AF | 1.18 (0.38-1.89) | 0.782 | 1.12 (0.41-1.30) | 0.976 |
| AF Duration (per 1 SD) | 1.08 (0.93-1.25) | 0.320 | 0.94 (0.85-1.03) | 0.179 |
| CHA2DS2Vasc Score (moderate risk vs. mild risk) | 1.07 (0.77-1.49) | 0.695 | 1.02 (0.83-1.25) | 0.874 |
| CHA2DS2Vasc Score (high risk vs. mild risk) | 0.76 (0.45-1.28) | 0.297 | 0.85 (0.62-1.15) | 0.291 |
| EUROScore (high risk vs. moderate or midl risk) | 0.96 (0.61-1.52) | 0.867 | 0.75 (0.55-1.02) | 0.065 |
| LAD (per 1 SD) | 0.96 (0.83-1.11) | 0.548 | 1.67 (1.03-2.12) | 0.017 |
| LVEDD (per 1 SD) | 0.99 (0.85-1.15) | 0.883 | 0.98 (0.90-1.07) | 0.697 |
| LVESD (per 1 SD) | 0.91 (0.76-1.09) | 0.285 | 0.96 (0.87-1.06) | 0.455 |
| LVEF (per 1 SD) | 0.97 (0.84-1.12) | 0.681 | 1.00 (0.91-1.09) | 0.926 |
| TAPSE (per 2 mm) | 1.04 (0.90-1.20) | 0.607 | 1.08 (0.99-1.18) | 0.086 |
| RA area (per 5 cm^2^) | 1.00 (0.85-1.18) | 0.956 | 1.03 (0.93-1.14) | 0.557 |
| TAD (per 4 mm) | 1.05 (0.90-1.23) | 0.536 | 1.04 (0.94-1.14) | 0.454 |
| TR severity (moderate vs. mid, no) | 0.79 (0.51-1.23) | 0.301 | 0.92 (0.70-1.20) | 0.531 |
| TR severtiy (high vs. mild, no) | 1.28 (0.81-2.03) | 0.288 | 1.16 (0.87-1.55) | 0.318 |
| PASP (per 15 mmHg) | 1.07 (0.83-1.39) | 0.595 | 1.39 (1.19-1.62) | ＜0.0001 |
| MV procedure (Replacement vs. repair) | 2.95 (2.09-4.17) | ＜0.0001 | 2.24 (1.84-2.73) | ＜0.0001 |
| CABG (yes vs. no) | 0.74 (0.33-1.68) | 0.473 | 0.98 (0.63-1.52) | 0.934 |
| TV repair (yes vs. no) | 1.32 (0.98-1.78) | 0.071 | 1.43 (1.19-1.72) | 0.00015 |
| CPB time (per 1 SD) | 1.09 (0.94-1.25) | 0.259 | 1.02 (0.93-1.11) | 0.725 |
| ACT (per 1 SD) | 1.09 (0.95-1.26) | 0.215 | 1.02 (0.93-1.11) | 0.662 |
| Increased or persistently increased PASP vs. persistently normal PASP | 8.11 (3.59-18.3) | ＜0.0001 | 61.1 (15.2-245) | ＜0.0001 |
| Increased or persistently increased PASP vs. decreased PASP | 5.96 (2.64-13.5) | ＜0.0001 | 1.33 (1.03-1.73) | ＜0.0001 |

ACT, aortic clamp time; AF, atrial fibrillation; BSA, body surface area; CABG, coronary artery bypss graft; CI, confidence interval; CPB, cardiopulmonary bypass; DMVD, degenerative mitral valve disease; HR, hazard ratio; LAD, left atrial diameter; LVEDD, left ventricular end diastolic diameter; LVEF, left ventricular ejection fraction; LVESD, left ventricular end systolic diameter; MV, mitral valve; PASP, pulmonary artery systolic pressure RA, right atrium; RMVD, rheumatic mitral valve disease; TAD, tricuspid annular diameter; TAPSE, tricuspid annular plane systolic excursion; TR, tricuspid regurgitation; TV, tricuspid valve; SD, standard deviation.

**Supplementary Figure Legends**

1. Kaplan-Meier analysis of survival according to the procedure type (repair vs. replacement) among the total cohort; (B) Kaplan-Meier analysis of survival according to the procedure type (repair vs. replacement) among patients with RMVD; (C) Kaplan-Meier analysis of survival according to the procedure type (repair vs. replacement) among patients with DMVD; MVR, mitral valve replacement; MVr, mitral valve repair; RMVD, rheumatic mitral valve disease; DMVD, degenerative mitral valve disease.
